# Unrecorded alcohol consumption in Russia: toxic denaturants and disinfectants pose additional risks

**DOI:** 10.2478/v10102-011-0030-x

**Published:** 2011-12

**Authors:** Yuriy V. Solodun, Yulia B. Monakhova, Thomas Kuballa, Andriy V. Samokhvalov, Jürgen Rehm, Dirk W. Lachenmeier

**Affiliations:** 1 Irkutsk Forensic Institute, Prosecutor General's Office, Shevzov Street 1, 664035 Irkutsk, Russia; 2 Chemisches und Veterinäruntersuchungsamt (CVUA) Karlsruhe, Weissenburger Strasse 3, D-76187 Karlsruhe, Germany; 3 Department of Chemistry, Saratov State University, Astrakhanskaya Street 83, 410012 Saratov, Russia; 4 Centre for Addiction and Mental Health (CAMH), 33 Russell Street, ARF 2035, Toronto, ON, M5S 2S1, Canada; 5 Department of Psychiatry, University of Toronto, 250 College Street, Toronto, ON, M5T 1R8, Canada; 6 Epidemiological Research Unit, Institute for Clinical Psychology and Psychotherapy, TU Dresden, Chemnitzer Strasse 46, D-01187 Dresden, Germany; 7 Dalla Lana School of Public Health, University of Toronto, 155 College Street, Toronto, ON, M5T 3M7, Canada

**Keywords:** alcohol, unrecorded alcohol, product quality, Russia, alcohol poisoning, risk assessment

## Abstract

In 2005, 30% of all alcohol consumption in Russia was unrecorded. This paper describes the chemical composition of unrecorded and low cost alcohol, including a toxicological evaluation. Alcohol products (n=22) from both recorded and unrecorded sources were obtained from three Russian cities (Saratov, Lipetsk and Irkutsk) and were chemically analyzed. Unrecorded alcohols included homemade samogons, medicinal alcohols and surrogate alcohols. Analysis included alcoholic strength, levels of volatile compounds (methanol, acetaldehyde, higher alcohols), ethyl carbamate, diethyl phthalate (DEP) and polyhexamethyleneguanidine hydrochloride (PHMG). Single samples showed contamination with DEP (275–1269 mg/l) and PHMG (515 mg/l) above levels of toxicological concern. Our detailed chemical analysis of Russian alcohols showed that the composition of vodka, samogon and medicinal alcohols generally did not raise major public health concerns other than for ethanol. It was shown, however, that concentration levels of DEP and PHMG in some surrogate alcohols make these samples unfit for human consumption as even moderate drinking would exceed acceptable daily intakes.

## Introduction

Alcohol consumption in Russia has been estimated to amount to 15.7 litres pure alcohol per adult capita in 2005, with most alcohol being consumed as spirits WHO (2011b); see also WHO Global Information System on Alcohol and Health (GISAH) (WHO (2011a)). Among drinkers, women are estimated to consume 16.3 l per capita, while men are estimated to consume considerably more per capita, at 35.4 l (WHO (2011a); own calculations). Overall, consumption was relatively stable in the past years with the 2008 estimate for adult per capita consumption amounting to 16.2 l, although roughly a third of this amount is considered to be unrecorded alcohol (Shield *et al.*, 2011). Unrecorded consumption in Russia not only includes illegally produced or smuggled spirits, but also legal non-beverage alcohols, such as medicinal tinctures, which are regularly consumed (Leon *et al.*, 2009). Unrecorded consumption by its very nature is hard to estimate, even though for Russia there is a long tradition of indirect estimation via alcohol poisoning and alcohol psychosis, which is also the basis of the estimates presented (Nemtsov, 1998, 2000).

Compared to many other countries, Russia has its own style of alcohol consumption with a rich history. The reasons for heavy drinking patterns and a high rate of alcohol use disorders in Russia (Rehm *et al.*, 2004, 2009) have been described as resulting from a complex set of economical, social, psychological and physiological factors (Zaigraev, 2009), the end result being that hazardous alcohol consumption is a social norm and is a part of culture and way of life for Russian people, especially in rural areas (Zaigraev, 2004).

The Russian Federation has a very low male life expectancy in comparison with other European countries (Zatonski *et al.*, 2008) and hazardous alcohol consumption is one of the key contributing factors (Gil *et al.*, 2010; Rehm *et al.*, 2007). The proportion of men who can be considered “binge drinkers” is relatively high at 40–50% (Bobak *et al.*, 2002; Popova *et al.*, 2007). Russians recognize the phenomenon of “zapoi”: a period of continuous drunkenness lasting several days in which the person is withdrawn from normal social life. As a result, negative consequences of alcohol consumption in Russia are much more prevalent than in the rest of Europe (Stickley *et al.*, 2009; Zaigraev, 2004). It has been estimated that alcohol may be responsible, directly or indirectly, for more than 30% of all deaths in Russia (Rehm *et al.*, 2007; Stickley *et al.*, 2009). According to Zaridze *et al. (*2009), the level of alcohol-attributable deaths contributing to premature mortality, particularly among men, is even higher – in their analyses, alcohol was a cause of more than half of all Russian deaths between the ages of 15–54 years. Other researchers estimate alcohol-related mortality to be between 170,000 (Leon *et al.*, 2009) to 750,000 (Nemtsov, 2002) people per year (which corresponds to an annual mortality rate of approximately 2.5 to 5.5 per 1,000). The most common causes of alcohol-associated deaths include unintentional and intentional injury including violence, alcohol poisoning, heart diseases and toxic hepatitis (Khaltourina & Korotayev, 2008; Razvodovsky, 2009; Solodun *et al.*, 2008).

Despite the fact that beverage preferences in large Russian cities have changed especially among young people during the last decade (Jargin, 2010), people from the older generation continue predominantly to buy and drink vodka (Zaigraev, 2009). Furthermore, especially in small towns and in rural regions, it is typical to consume unrecorded beverages (predominantly samogon) and surrogate alcohols (substances that are not intended for human consumption) including medicinal alcohol, aftershave and other lotions, perfume, antifreeze, brake fluid, denatured alcohol, glues, gasoline, kerosene, tooth powder, and vinegar (Zaigraev, 2004). Even in big cities such as Izhevsk, drinking of unrecorded alcohols was relatively common in 2003–2005 (Pridemore *et al.*, 2010). The main reasons for drinking surrogates are the high affordability and physical availability of surrogates because they have a unit cost for ethanol below that of standard Russian vodka (Bobrova *et al.*, 2009; Gil *et al.*, 2009).

In Russia several cases of mass poisoning by alcohol-containing liquids were recorded during the last decade (Tsisanova & Salomatin, 2010). The first case was registered in Yekaterinburg (Siberia) in 2004, and further reports spread among the 21 regions throughout Russia during the following years. Surrogate alcohols (disinfectants, medicinal alcohols, perfumes) from unidentified sources were consumed in all cases, with the chief general manifestation being the development of toxic hepatitis. Toxicological characteristics of the consumed unrecorded alcohols have not been systematically studied so far.

There are several papers that deal with the investigation of the composition of vodkas and unrecorded beverages from Russia. In the largest survey of Russian unrecorded alcohols done by Nuzhnyi (2004), 81 samples of samogons were analyzed. In this study most of the samples investigated were similar to commercial spirits. Savchuk *et al.* (2006) studied 13 illegally produced strong alcoholic beverages from the Russian cities of Kyzyl and Stavropol in which diethyl phthalate (DEP) was found, indicative of the fact that denatured alcohol was used to produce these beverages. In one sample ethylene glycol was found, probably due to the fact that the sample was poured in a plastic bottle with residual amounts of a technical liquid containing this compound. However, the authors came to the conclusion that the specified amount of DEP and ethylene glycol would not increase the acute toxicity of ethanol. The general conclusion to be drawn is that the great majority of the alcohol-containing liquids from Russia studied so far were close to commercial alcoholic beverages in terms of chemical composition and toxic properties (Nuzhnyi, 2004; Savchuk *et al.*, 2006). Long-term toxicological studies of the alcohol surrogates clearly indicated that the toxicity of alcohol surrogates was similar to that of legal high-quality vodka or other beverages with the same percentage of alcohol in the great majority of cases. These materials fully satisfied the requirements of the Russian State Standard GOST (2006).

Our goal is therefore to identify potentially toxic impurity components in alcoholic beverages and various alcohol-containing liquids from the Russian market. To reveal the potentially toxic properties of unrecorded alcohols in our study, we compared the composition of beverage alcohol (vodka and samogon) with surrogate alcohol (medicinal and denatured alcohols).

## Materials and methods

### Sampling

Samples of the alcoholic beverages were bought from individual producers (samogon) and local drug stores (medicinal alcohol) in Saratov and Lipetsk – middle-sized Russian cities situated in the European part of Russia. Nine samples that were withdrawn from circulation in the course of state monitoring of alcohol marketing in Irkutsk (Central Siberia) were also examined. Four vodka samples were legally bought in shops and were used as reference samples.

We collected and analyzed these types of unrecorded alcohols because they have traditionally been preferred beverages in Eastern Europe (Popova *et al.*, 2007). A similar sampling strategy was used in the course of analysis of Ukraine samples (Lachenmeier *et al.*, 2010). Table 1 gives an overview about the category and origin of the samples.

**Table 1:** Sample description of alcohol products from the Russian market.

Code	Product	Manufacturer	Point of sale	Ethanol source	Labelling
Y1	Samogon	home made	Saratov	Fruits	40% vol
Y2	Samogon	home made	Saratov	Fruits	40% vol
Y3	Samogon	home made	Saratov	Fruits	40% vol
Y4	Samogon	home made	Saratov	Fruits	40% vol
Y5	Samogon	home made	Saratov	(unknown)	40% vol
Y6	Samogon	home made	Saratov	(unknown)	40% vol
Y7	Vodka “Russkay bereza”	“Moj Stolitca”	Saratov	Grain	40% vol
Y8	Vodka “Metelitca ”	“Sadko”	Saratov	Grain	40% vol
Y9	Medicinal alcohol (haw tincture)	“Gippocrat”	Saratov	(unknown)	70% vol
Y10	Medicinal alcohol (haw tincture)	“Gippocrat”	Saratov	(unknown)	70% vol
N1	Samogon	home made	Lipetsk	(unknown)	no label
N2	Vodka “Janskaj”	“Topaz”	Lipetsk	Sugar	40% vol
N3	Vodka “Voronezkaj”	“Kratos”	Lipetsk	Grain	40% vol
S1	Surrogate alcohol	(unknown)	Irkutsk	(unknown)	no label
S2	Surrogate alcohol	(unknown)	Irkutsk	(unknown)	no label
S3	Surrogate alcohol	(unknown)	Irkutsk	(unknown)	no label
S4	Surrogate alcohol	(unknown)	Irkutsk	(unknown)	no label
S5	Medicinal alcohol	(unknown)	Irkutsk	(unknown)	no label
S6	Medicinal alcohol	(unknown)	Irkutsk	(unknown)	no label
S7	Medicinal alcohol	(unknown)	Irkutsk	(unknown)	no label
S8	Medicinal alcohol	(unknown)	Irkutsk	(unknown)	no label
S9	Medicinal alcohol (haw tincture)	(unknown)	Irkutsk	(unknown)	no label

### Analytical procedure

The analytical methodology was similar to the one used in previous studies in Central and Eastern Europe (Lachenmeier *et al.*, 2009a, b Lachenmeier *et al.*, 2010). In brief, alcoholic strength was determined by Fourier transform infrared spectroscopy (Lachenmeier, 2007a). Volatile components were analyzed on the basis of the European Union Reference Methods for the Analysis of Spirits using gas chromatography (GC) with a flame-ionization detector (FID) (European Commission, 2000; Lachenmeier *et al.*, 2006). Ethyl carbamate (urethane) was determined using GC with tandem mass spectrometry (GC-MS/MS) (Lachenmeier *et al.*, 2005). Furthermore, all samples were screened for unknown substances using nuclear magnetic resonance spectroscopy (NMR) similar to the procedure in Lachenmeier *et al.* (2009b). NMR was also used to quantify diethyl phthalate (DEP). For the polyhexamethyleneguanidine hydrochloride (PHMG) quantification we applied a spectroscopic procedure with Eosin Y as indicator was applied (Chmilenko *et al.*, 2010). The full methodology for screening and quantification of DEP and PHMG is available in Monakhova *et al.* (2011).

### Toxicological evaluation

The toxicological evaluation of many compounds in alcoholic beverages is problematic, since even for the most common compounds such as higher alcohols no European or international maximum limits have been established. This paper therefore uses the criteria established by the Alcohol Measures for Public Health Research Alliance (AMPHORA) project, which are generally based on acceptable daily intakes (ADI) for foods with the assumption of a lifetime daily exposure. A detailed rationale for the limits proposed by AMPHORA was previously published (Lachenmeier *et al.*, 2011b). Furthermore, we used the Russian State Standard (GOST, 2006) for rectified ethyl alcohol for comparison.

## Results

A total of 22 samples were collected and analyzed. Table 2 presents the results of the analysis of all samples. The ethanol content in beverages varied between 26.2% vol to 81.4% vol. The highest alcoholic strengths were typically found in medicinal alcohol from pharmacies and in surrogate alcohols (typically around 70% vol), while the vodkas had a very uniform alcoholic strength around 40% vol. The homemade samogons showed a higher variation in their alcoholic strengths, with a mean value at around 32% vol (26.2–46.7% vol range).

**Table 2:** Volatile composition of Russian alcohol products. Values are given in g/hl pa (with the exception of ethanol [% vol], ethyl carbamate, PHMG and diethyl phthalate (DEP) [mg/l]) *

Category	Code	Ethanol [% vol]	Acetaldehyde	Methanol	1-Propanol	1-Butanol	Iso-Butanol	Amyl alcohols	1-Hexanol	2-Phenyl ethanol	Sum of higher alcohols	Ethyl acetate	Benzyl acetate	Ethyl lactate	Ethyl caprylate	Ethyl benzoate	Benzaldehyde	Ethyl carbamate [mg/l]	DEP	PHMG
Sam.^a^	Y1	26.2	13.2	118	29.9	0.3	53.0	191	n.d.^e^	9.8	284	22.6	n.d.	20.2	n.d.	n.d.	n.d.	n.d.	n.d.	n.d.
Sam.	Y2	35.6	10.9	325	21.4	2.7	55.4	201	1.8	4.4	286	101	n.d.	50.0	n.d.	n.d.	n.d.	0.06	n.d.	n.d.
Sam.	Y3	40.9	23.1	5.6	6.3	n.d.	179	282	n.d.	18.3	485	n.d.	21.0	n.d.	n.d.	n.d.	n.d.	0.06	n.d.	n.d.
Sam.	Y4	43.0	9.8	67.2	31.2	1.1	41.3	148	n.d.	2.9	225	87.0	n.d.	211	1.1	n.d.	n.d.	0.16	n.d.	n.d.
Sam.	Y5	46.7	16.3	1.9	13.5	n.d.	92.5	163	n.d.	7.8	277	n.d.	18.4	n.d.	n.d.	n.d.	n.d.	n.d.	n.d.	n.d.
Sam.	Y6	29.8	29.4	216	49.8	1.1	87.8	333	n.d.	9.9	482	51.3	n.d.	30.1	n.d.	n.d.	n.d.	0.06	n.d.	n.d.
Vod.^b^	Y7	40.1	n.d.	2.3	n.d.	n.d.	n.d.	n.d.	n.d.	n.d.	n.d.	n.d.	n.d.	n.d.	n.d.	n.d.	n.d.	n.d.	n.d.	n.d.
Vod.	Y8	40.1	0.5	5.9	n.d.	n.d.	n.d.	n.d.	n.d.	n.d.	n.d.	n.d.	n.d.	n.d.	n.d.	n.d.	n.d.	n.d.	n.d.	n.d.
Med.^c^	Y9	**	1.2	4.3	n.d.	n.d.	n.d.	n.d.	n.d.	n.d.	n.d.	1.6	n.d.	n.d.	n.d.	n.d.	n.d.	n.d.	n.d.	n.d.
Med.	Y10	**	1.2	7.4	n.d.	n.d.	n.d.	n.d.	n.d.	n.d.	n.d.	n.d.	n.d.	n.d.	n.d.	n.d.	n.d.	n.d.	n.d.	n.d.
Sam.	N1	40.6	19.1	3.4	35.1	1.2	162	242	n.d.	10.6	451	28.7	n.d.	1.2	1.0	n.d.	n.d.	n.d.	n.d.	n.d.
Vod.	N2	40.2	0.6	2.3	n.d.	n.d.	n.d.	n.d.	n.d.	n.d.	n.d.	n.d.	n.d.	n.d.	n.d.	n.d.	n.d.	n.d.	n.d.	n.d.
Vod.	N3	40.1	0.6	4.0	n.d.	n.d.	n.d.	n.d.	n.d.	n.d.	n.d.	n.d.	n.d.	n.d.	n.d.	n.d.	n.d.	n.d.	n.d.	n.d.
Sur.^d^	S1	75.3	0.7	2.9	n.d.	n.d.	n.d.	n.d.	n.d.	n.d.	n.d.	n.d.	n.d.	n.d.	n.d.	n.d.	n.d.	n.d.	1269	515
Sur.	S2	30.7	20.5	817	n.d.	n.d.	n.d.	1.5	n.d.	n.d.	1.5	2.9	n.d.	n.d.	n.d.	n.d.	n.d.	0.06	n.d.	n.d.
Sur.	S3	81.4	0.5	3.6	n.d.	n.d.	n.d.	n.d.	n.d.	n.d.	n.d.	n.d.	n.d.	n.d.	n.d.	n.d.	n.d.	n.d.	n.d.	n.d.
Sur.	S4	79.7	1.9	10.0	n.d.	n.d.	n.d.	n.d.	n.d.	n.d.	n.d.	1.6	n.d.	n.d.	n.d.	n.d.	n.d.	n.d.	n.d.	n.d.
Med.	S5	**	6.1	9.9	n.d.	n.d.	n.d.	n.d.	n.d.	n.d.	n.d.	1.0	n.d.	n.d.	n.d.	3.1	0.6	n.d.	n.d.	n.d.
Med.	S6	72.1	0.7	3.0	n.d.	n.d.	n.d.	n.d.	n.d.	n.d.	n.d.	n.d.	n.d.	n.d.	n.d.	n.d.	n.d.	n.d.	n.d.	n.d.
Med.	S7	69.8	5.2	3.7	n.d.	n.d.	n.d.	n.d.	n.d.	n.d.	n.d.	1.4	n.d.	n.d.	n.d.	n.d.	n.d.	n.d.	n.d.	n.d.
Med.	S8	67.2	2.5	11.7	n.d.	n.d.	n.d.	n.d.	n.d.	n.d.	n.d.	1.6	n.d.	n.d.	n.d.	n.d.	n.d.	n.d.	n.d.	n.d.
Med.	S9	80.0	0.7	2.9	n.d.	n.d.	n.d.	n.d.	n.d.	n.d.	n.d.	n.d.	n.d.	n.d.	n.d.	n.d.	n.d.	n.d.	275	n.d.
AMPHORA limit		–	50	1000	–	–	–	–	–	–	1000	1000	–	–	–	–	500	0.4	480	–
Russian GOST		–	0.01	0.4	–	–	–	–	–	–	0.035	0.030^f^	–	–	–	–	–	–	–	–

a Samogon

b Vodka

c Medicinal alcohol

d Surrogate alcohol

e n.d.: not detected (detection limit 0.5 g/hlpa); negative in all samples: benzyl alcohol, 2-butanol, methyl acetate

f Sum of all esters

* all measurements were done in triplicate, the mean values are shown

** not enough sample amount for Fourier transform infrared (FTIR) spectroscopic measurement

Methanol was detected in concentrations ranging from undetectable to 817 g/hl of pure alcohol (g/hlpa). Generally, the methanol levels were within acceptable ranges below 10 g/hl pa, with some exceptions (Y1, Y2, Y4, Y6, S2, S8). The content of methanol in one surrogate alcohol (S2) was 817 g/hlpa. The quantities of other volatile compounds (acetaldehyde, ethyl acetate and higher alcohols) in the samples were higher than in the commercial vodkas but still acceptable. All the samples tested had very low or even non detectable ethyl carbamate content according to GC-MS/MS analysis. During the targeted NMR analysis, DEP was found in two of the samples investigated (surrogate alcohol S1 and medicinal alcohol S9) in concentrations of 1,269 and 275 mg/l, respectively. Furthermore, we detected the presence of PHMG in one sample (S1), with the concentration of 515 mg/l.

## Discussion

### Alcoholic strength

The alcohol content of samogon and vodka from Russia varies generally between 26% vol and 47% vol (average 39% vol). This is in contrast to other studies in Central and Eastern Europe, in which unrecorded alcohol contained higher alcoholic strengths than recorded alcohol. For example, in Poland, the unrecorded spirits typically contained around 48% vol with some products as high as 85% vol (Lachenmeier *et al.*, 2009a). However, alcoholic strength of our samples was found to be similar to the typical strength of unrecorded beverages from Ukraine (the majority of these samples had a uniform alcohol content around 40% vol) (Lachenmeier *et al.*, 2010) and to other investigations of Russian unrecorded beverages (McKee *et al.*, 2005; Nuzhnyi, 2004). For Russia, we can conclude that ethanol in samogon would probably cause similar effects (*i.e.* regarding intoxication and chronic effects) as recorded spirits.

Medicinal and surrogate alcohol contained 70% vol of alcohol on average. Similarly, McKee *et al.* (2005) found that medicinal alcohol contained 66% vol and Lang *et al.* (2006) observed that medicines used as surrogate alcohol had 67% vol. Likewise, the alcoholic strength of Ukraine medicinal alcohol was found to be 69% vol on average (Lachenmeier *et al.*, 2010). Thus, the finding of comparably high-strength alcohol in medicinal unrecorded alcohol is consistent throughout the countries of the former Soviet Union. In the light of the fact that it is possible to legally purchase many different non-beverage alcohol products with at least 60% ethanol content (Gil *et al.*, 2009), consuming these comparably high-strength alcohols seems to be of public health relevance in the Russian Federation.

### Volatile composition and ethyl carbamate

Besides ethanol, unrecorded alcohols from Russia contained several volatile compounds formed in the process of alcoholic fermentation. For example, methanol is the substance most often blamed for lethal alcohol poisonings (Lachenmeier *et al.*, 2007b). However, the methanol content in our Russian samples was relatively low (*i.e.* lower than the EU limit of 30 g/hl pa for neutral alcohol (European Parliament and Council, 2008) and comparable with the Ukraine study where the average content of methanol was 9.8 g/hl pa (Lachenmeier *et al.*, 2010). Moreover, the methanol content was lower than that found in recent studies of unrecorded alcohols from Poland (Lachenmeier *et al.*, 2009a), Lithuania and Hungary (Lachenmeier *et al.*, 2009c). Samples S2, Y2 and Y6 are the three exceptions with a relatively high methanol content (more than 200 g/hl pa). This may be explained by the use of fruits as the base component (apples and pears), which naturally contain methanol precursors (Lachenmeier & Musshoff, 2004). For this reason, the methanol limit for fruit spirits in the EU is set at 1000 g/hl pa (which equates to 0.4% vol methanol at 40% vol alcohol) (European Parliament and Council, 2008). None of the samples exceeded this limit, suggesting that methanol content did not pose a threat to public health.

Acetaldehyde associated with alcohol consumption is regarded as ‘carcinogenic to humans’ (IARC Group1) (Secretan *et al.*, 2009). Due to the fact that complete separation of acetaldehyde is not technically possible (at least not for home producers), acetaldehyde was found in all home-produced spirits (the average value was 17 g/hl pa). In commercial vodka very low acetaldehyde concentrations were found (below 1 g/hl pa). None of the samples exceeded the AMPHORA limit of 50 g/hl pa for acetaldehyde.

Components of fusel oil (propanol, butanol, isoamyl alcohol, hexanol) were found in samogon samples with an average concentration of 356 g/hl pa. This is consistent with previous investigations of home-produced spirits (Huckenbeck *et al.*, 2003; Lachenmeier *et al.*, 2009a, c, Lachenmeier *et al.*, 2010; Lang *et al.*, 2006; Szücs *et al.*, 2005) and samogons in Russia (Nuzhnyi 2004). However, the AMPHORA limit of 1,000 g/hl pa was exceeded by none of the samples from Russia. Ethyl carbamate (urethane) was detected in 5 samples with the average concentration below 0.09 mg/l, which is even lower than in samples from Ukraine (Lachenmeier *et al.*, 2010). All other analyzed compounds listed in Table 2 were not detectable or below the AMPHORA limits.

None of the samples in this targeted analysis exceeded the AMPHORA limits for substances listed in Table 2. However, the levels of methanol and acetaldehyde in the vodkas exceeded the Russian regulations (GOST, 2006). It must, however, be mentioned that this regulation appears to be guided by quality considerations, so the limits are not based on toxicological thresholds but on ‘best practices’ or levels desirable from an organoleptic standpoint. For example, the methanol limit in Europe is 10 g/hl pa for vodka (European Parliament and Council 2008). In Russia this limit is 0.4 g/hl pa, which is lower than the maximum tolerable concentration of 5,000 g/hl pa (Paine & Dayan, 2001) by a factor of 12,500. Therefore, Russian laws provide a large margin of safety, and exceeding these limits cannot be interpreted directly as a public health problem.

### Diethyl phthalate

DEP was detected in two samples in amounts (275 and 1269 mg/L) comparable with those found in Russian samples from Kyzyl (Savchuk *et al.*, 2007) and Lithuanian unrecorded alcohols (Lachenmeier *et al.* 2009c). DEP was a common denaturing agent for ethanol and alcohol-containing products in Russia before 2006 (Savchuk *et al.*, 2007; Tsisanova & Salomatin, 2010). Now the list of denaturants in Russia specified by the Federal Law No 171 from July 27, 2010 for all kinds of alcoholic products includes kerosin and gasoline (no less than 0.5% vol), bitrex (no less than 0.0015% mas), crotonaldehyde (no less than 0.2% vol) (Federal Law, 2010) but DEP was excluded from this list. However, DEP can still be occasionally found in surrogate alcohols (Solodun *et al.*, 2008; Tsisanova & Salomatin, 2010) and should be considered as a toxic contaminant in such products.

An accepted toxicological threshold for oral exposure to DEP is missing. An oral reference dose (RfD) of DEP was set by the US Environmental Protection Agency (EPA) (US EPA, 2003) at 0.8 mg/kg bodyweight/day (48 mg/day for a 60-kg human) with extrapolation from the short-term animal toxicity experiments of Brown *et al.* (1978). The WHO (2003) estimated a tolerable daily intake (TDI) of 5 mg/kg bodyweight (300 mg/day for a 60-kg-human) from a NOAEL (No observed adverse effect level) of 1,600 mg/kg bodyweight for developmental effects, derived from the same study of Brown *et al.* (1978). Table 3 presents different exposure scenarios for drinkers of alcohols contaminated with DEP. The US EPA RfD limit is exceeded by 3 or more drinks per day for surrogate alcohol (S1). However, drinking the contaminated medicinal alcohol (S9) would not yet exceed the US EPA RfD. To assess the average risk of DEP consumption with surrogate alcohols, we calculated the DEP exposure for average and worst case (95^th^ percentile) scenarios with the combined data available in the literature (Savchuk *et al.*, 2006) and from our study. According to the results obtained, daily consumption of 2 standard drinks (which is considered light consumption in Russia (Djdichko & Evdokimova, 2008)) poses a health risk as the US EPA RfD is exceeded. For binge drinkers the WHO TDI level may be exceeded after 7 drinks per day in the worst case scenario.

**Table 3:** Exposure of DEP and PHMG in different Russian consumption scenarios

Exposure scenarios for drinkers of contaminated surrogate alcohol					
	Exposure DEP^a^ (mg/kg bodyweight/ day)				Exposure PHMG^a^ (mg/kg bodyweight/day)
Number of daily drinks^b^	S1	S9	Average^c^	P95^c^	S1
1	0.4	0.1	0.4	0.7	0.07^f^
2	0.7	0.2	0.9^d^	1^d^	0.1^f^
3	1^d^	0.2	1^d^	2^d^	0.2^f^
5	2^d^	0.4	2^d^	4^d^	0.4^f^
7	3^d^	0.5	3^d^	5^e^, ^d^	0.5^f^
10	4^d^	0.7	4^d^	7^e^, ^d^	0.7^f^

a Exposure=((12.67 ml) * (DEP/PHMG concentration [mg/l]) * (Number of standard drinks) * 100)/((alcohol strength [% vol]) * 1000 * 60 kg)

b A standard drink in Russia is considered to have a total of 12.67 ml of pure alcohol (Djdichko & Evdokimova, 2008)

c Calculated with the combined values of the Savchuk *et al.* (2006) study (1022, 1284, 964, 1270, 850, 1073 mg/l) and our study (1269, 275 mg/l)

d Value exceeds US EPA RfD threshold (0.8 mg/kg bodyweight/ day) (US EPA, 2003)

e Value exceeds WHO TDI threshold (5 mg/kg bodyweight/ day) (WHO, 2003)

f Value exceeds provisional TDI (0.5 g/kg bodyweight/day) extrapolated from the study of Condrashov (1992)

### Polyhexamethyleneguanidine hydrochloride (PHMG)

Besides diethyl phthalate, polyhexamethyleneguanidine hydrochloride was detected in one of the surrogate alcohols (515 mg/l) (S1). PHMG is an effective antiseptic and is commonly used for suppression of hospital infection in the Russian Federation (Tsisanova & Salomatin, 2010). PHMG (0.10–0.14%) together with DEP (0.08–0.15%) were contained in disinfectants that were used as an ethanol source in several poisoning cases in Russia (Tsisanova & Salomatin, 2010). As commercial preparation of disinfectants usually contain PHMG in concentrations of around 1,000 mg/l, it can be assumed that our sample was a disinfectant diluted 1:1 with water and/or alcohol of other sources.

For toxicological evaluation of PHMG, only limited human data is available. It has been assumed that consumption of surrogate alcohol containing PHMG induces significant disorders of lipid metabolism, which ultimately may lead to liver injuries, particularly toxic hepatitis (Makarov & Ryasenskii, 2009). On the basis of clinical manifestations and laboratory findings of 579 poisoned patients, Ostapenko *et al.* (2011) concluded that cholestatic hepatitis was caused by PHMG-containing alcohol, while a history of alcohol-induced hepatitis and cirrhosis contributed to a more severe course of the poisoning. However, neither of the two studies provide clear evidence how the authors in both studies distinguished between the effects of PHMG and ethanol, which of course may also cause acute and chronic liver injury (Lieber 1988; Rehm *et al.*, 2010). Therefore, only animal experiments can be taken as a basis for risk assessment of PHMG. The LD_50_ for PHMG was found to be 450 mg/kg for mice and 630 mg/kg for rats (Condrashov, 1992). In these experiments, liver, spleen and stomach injuries were reported. The NOAEL in a 6-month oral study with rats was found to be 0.1 mg/kg bodyweight/day by Condrashov (1992). The animals in the 1.0 mg/kg bodyweight/day and 10 mg/kg bodyweight/day dose groups showed an increase in liver and spleen weights and also changes in blood enzyme levels. In another study (Yushkov *et al.*, 2011), single doses of 50 mg/kg/day were introduced intraperitoneally to white rats. The rats were sacrificed after 1, 2, 3 and 7days. Blood analysis revealed acute inflammation (high levels of granulocytes, eosinophils, erythrocyte sedimentation rate, and others) and development of toxic hepatitis (high levels of bilirubin and aspartate aminotransferase) in two to three days after introduction of PHMG. Besides liver effects, PHMG induced general toxic changes in the kidney and pancreas, and suppressed the immune system.

No oral long-term study was available, which is normally used to extrapolate from animals to humans. To make a first judgment about the risk of PHMG in the alcohols, we decided to use a provisional TDI of 0.5 µg/kg bodyweight/day (0.03 mg/day for a 60-kg human) extrapolated from the animal NOAEL of 0.1 mg/kg bodyweight/day with an uncertainty factor of 200 (*i.e.* the standard uncertainty factor of 100 for extrapolation from animal experiments to humans, and an additional factor of 2 to consider the 6-month study) (Lachenmeier *et al.*, 2011a). With this in mind, 0.05 ml of our surrogate alcohol containing PHMG could exceed this toxicological threshold for a 60-kg person. In other words, the TDI level is exceeded after consuming only one standard drink of surrogate alcohol with PHMG (Table 3). It is therefore plausible that in regions where disinfectants with PHMG were consumed, high levels of toxic hepatitis, histologically different from chronic hepatitis induced by long-term ethanol consumption, were recorded (Ostapenko *et al.*, 2011; Solodun *et al.*, 2008; Tsisanova & Salomatin, 2010). According to our own histological observations from *post mortem* cases, PHMG intoxication causes fulminant granulomatous inflammation, which fails to complete because of the patient's death and there are no signs of liver cirrhosis typical for ethanol poisoning. While this observation is plausible regarding the available evidence, it must be noted that the actual exposure in the *post mortem* cases is unclear.

### Recommendations and alcohol policy aspects

Alcohol-containing liquids based on surrogate alcohols are widespread on the illicit market (Tsisanova & Salomatin, 2010). We found that some of the samples studied contained relatively high concentrations of DEP and PHMG. Such products could have been a common source of ethanol among the low-income Russian population due to their availability and low cost (Solodun *et al.*, 2008). However, a larger number of samples is clearly needed to evaluate the magnitude of the problem on a population scale. The sale of such products is usually carried out by means of illegal bottling in standard vodka package (Solodun *et al.*, 2008). Currently, manufacturing and sale of disinfectants with PHMG and DEP are suspended. However the problem apparently still exists, probably because of supplies existing in storage that cannot be sold. Thus, continued attempts to control and to reduce the availability of nonbeverage alcohols should be a public health priority in Russia (Lachenmeier *et al.*, 2011c). We expect that the incidence could decline in the future due to the legislative changes. The composition of samogon and medicinal alcohol in our samples is not substantially different from the same products from other European countries and is close to commercial alcoholic beverages in terms of toxic properties. Other aspects for alcohol policy in Russia can be pointed out. Besides generally recommended policy measures such as taxation and availability restrictions (Babor *et al.*, 2010; Rehm *et al.*, 2008), it might be useful to attentively control the composition of denatured alcohols and consumer products for substances that are prohibited by law but still are in circulation. Overall, there have been efforts to reduce unrecorded consumption in Russia, and these measures seem to have been successful, based on official Russian statistics and on indirect estimation (Nemtsov, personal communication). However, the level of unrecorded consumption is still comparatively high in Russia. From a public health point of view, two measures seem to be necessary in future which would contribute to an overall reduction of consumption and alcohol-attributable harm (for details of mechanisms see Babor *et al.* (2010)):

- Reduce the number of heavy drinking occasions.
- Further reduce the level of unrecorded consumption.

## References

[CIT0001] Babor T, Caetano R, Casswell S, Edwards G, Giesbrecht N, Graham K, Grube J, Hill L, Holder H, Homel R, Livingstone M, Österberg E, Rehm J, Room R, Rossow I (2010). Alcohol: No ordinary commodity. Research and public policy.

[CIT0002] Bobak M, McKee M, Rose R, Marmot M (2002). Alcohol consumption in a national sample of the Russian population. Addiction.

[CIT0003] Bobrova N, West R, Malutina D, Koshkina E, Terkulov R, Bobak M (2009). Drinking alcohol surrogates among clients of an alcohol-misuser treatment clinic in Novosibirsk, Russia. Subst Use Misuse.

[CIT0004] Brown D, Butterworth KR, Gaunt IF, Grasso P, Gangolli SD (1978). Short-term oral toxicity study of diethyl phthalate in rat. Food Cosmet Toxicol.

[CIT0005] Chmilenko TS, Galimbievskay EA, Chmilenko FA (2010). Formation of bromophenol red ion associates and their interaction with polyhexamethyleneguanidine in water solutions. Met Obj Chim Anal.

[CIT0006] Condrashov SA (1992). Hygienic evaluation of new polymer flocculant polyhexamethyleneguanidine. Gig Sanit.

[CIT0007] Djdichko A, Evdokimova E (2008). Moderate consumption: where are the limits?. Med Gaz.

[CIT0008] European Commission (2000). Commission Regulation (EC) No 2870/2000 laying down Community reference methods for the analysis of spirits drinks. Off J Europ Comm.

[CIT0009] European Parliament and Council (2008). Regulation (EC) No 110/2008 of the European Parliament and of the Council of 15 January 2008 on the definition, description, presentation, labelling and the protection of geographical indications of spirit drinks and repealing Council Regulation (EEC) No 1576/89. Off J Europ Union.

[CIT0010] Federal Law (2010). No. 171. About performing of state control of production and turnover of ethyl alcohol and alcohol products from 27.07.2010.

[CIT0011] Gil A, Polikina O, Koroleva N, Leon DA, McKee M (2010). Alcohol policy in a Russian region: a stakeholder analysis. Eur J Pub Health.

[CIT0012] Gil A, Polikina O, Koroleva N, McKee M, Tomkins S, Leon DA (2009). Availability and characteristics of nonbeverage alcohols sold in 17 Russian cities in 2007. Alcohol Clin Exp Res.

[CIT0013] GOST (2006). Russian State Standard R 51652-2000. Rectified ethyl alcohol of food raw material. Specifications.

[CIT0014] Huckenbeck W, Freudenstein P, Jeszenszky E, Scheil HG (2003). Congeners in spirits produced by moonshine distillers. Blutalkohol.

[CIT0015] Jargin S (2010). Letter from Russia: minimal price for vodka established in Russia from 1 January 2010. Alcohol Alcohol.

[CIT0016] Khaltourina DA, Korotayev AV (2008). Potential for alcohol policy to decrease the mortality crisis in Russia. Eval Health Prof.

[CIT0017] Lachenmeier DW (2007a). Rapid quality control of spirit drinks and beer using multivariate data analysis of Fourier transform infrared spectra. Food Chem.

[CIT0018] Lachenmeier DW, Frank W, Kuballa T (2005). Application of tandem mass spectrometry combined with gas chromatography to the routine analysis of ethyl carbamate in stone-fruit spirits. Rapid Commun Mass Spectrom.

[CIT0019] Lachenmeier DW, Ganss S, Rychlak B, Rehm J, Sulkowska U, Skiba M, Zatonski W (2009a). Association between quality of cheap and unrecorded alcohol products and public health consequences in Poland. Alcohol Clin Exp Res.

[CIT0020] Lachenmeier DW, Humpfer E, Fang F, Schütz B, Dvortsak P, Sproll C, Spraul M (2009b). NMR-spectroscopy for nontargeted screening and simultaneous uantification of health-relevant compounds in foods: The Example of Melamine. J Agric Food Chem.

[CIT0021] Lachenmeier DW, Monakhova YB, Samokhvalov AV, Rehm J (2011a). Causality between polyhexamethylene-guanidine occurance in unrecorded alcohol and cholestatic hepatitis outbreak in Russia. Clin Toxicol.

[CIT0022] Lachenmeier DW, Musshoff F (2004). Volatile congeners in alcoholic beverages. Retrospective trends, batch comparisons and current concentration ranges. Rechtsmed.

[CIT0023] Lachenmeier DW, Rehm J, Gmel G (2007b). Surrogate alcohol: what do we know and where do we go?. Alcohol Clin Exp Res.

[CIT0024] Lachenmeier DW, Samokhvalov AV, Leitz J, Schoeberl K, Kuballa T, Linskiy IV, Minko OI, Rehm J (2010). The composition of unrecorded alcohol from eastern Ukraine: Is there a toxicological concern beyond ethanol alone?. Food Chem Toxicol.

[CIT0025] Lachenmeier DW, Sarsh B, Rehm J (2009c). The composition of alcohol products from markets in Lithuania and Hungary, and potential health consequences: A pilot study. Alcohol Alcohol.

[CIT0026] Lachenmeier DW, Schoeberl K, Kanteres F, Kuballa T, Sohnius E-M, Rehm J (2011b). Is contaminated unrecorded alcohol a health problem in the European Union? A review of existing and methodological outline for future studies. Addiction.

[CIT0027] Lachenmeier DW, Sohnius E-M, Attig R, López MG (2006). Quantification of selected volatile constituents and anions in Mexican Agave spirits (Tequila, Mezcal, Sotol, Bacanora). J Agric Food Chem.

[CIT0028] Lachenmeier DW, Taylor B, Rehm J (2011c). Alcohol under the radar: Do we have policy options regarding unrecorded alcohol?. Int J Drug Policy.

[CIT0029] Lang K, Väli M, Szücs S, Ádány R, McKee M (2006). The composition of surrogate and illegal alcohol products in Estonia. Alcohol Alcohol.

[CIT0030] Leon DA, Shkolnikov VM, McKee M (2009). Alcohol and Russian mortality: A continuing crisis. Addiction.

[CIT0031] Lieber CS (1988). Biochemical and molecular basis of alcohol-induced injury to liver and other tissues. N Engl J Med.

[CIT0032] Makarov VK, Ryasenskii DS (2009). Assessments of effects produced by taking in polymethyleneguanidine hydrochloride on lipid composition of blood serum. Toksikol Vest.

[CIT0033] McKee M, Süzcs S, Sárváry A, Ádany R, Kiryanov N, Saburova L, Tomkins S, Andreev E, Leon DA (2005). The composition of surrogate alcohols consumed in Russia. Alcohol Clin Exp Res.

[CIT0034] Monakhova YB, Kuballa T, Leitz J, Lachenmeier DW (2011). Determination of diethyl phthalate and polyhexamethylene guanidine in surrogate alcohol from Russia. Int J Anal Chem.

[CIT0035] Nemtsov AV (1998). Alcohol-related harm and alcohol consumption in Moscow before, during and after a major anti-alcohol campaign. Addiction.

[CIT0036] Nemtsov AV (2000). Estimates of total alcohol consumption in Russia, 1980–1994. Drug Alcohol Depend.

[CIT0037] Nemtsov (2002). Alcohol-related human losses in Russia in the 1980s and 1990s. Addiction.

[CIT0038] Nuzhnyi V, Haworth A, Simpson R (2004). Chemical composition, toxic, and organoleptic properties of noncommercial alcohol samples. Moonshine Markets. Issues in unrecorded alcohol beverage production and consumption.

[CIT0039] Ostapenko YN, Brusin KM, Zobnin YV, Shchupak AY, Vishnevetskiy MK, Sentsov VG, Novikova OV, Alekseenko SA, Lebed'ko OA, Puchkov YB (2011). Acute cholestatic liver injury caused by polyhexamethyleneguanidine hydrochloride admixed to ethyl alcohol. Clin Toxicol.

[CIT0040] Paine AJ, Dayan AD (2001). Defining a tolerable concentration of methanol in alcoholic drinks. Hum Exp Toxicol.

[CIT0041] Popova S, Rehm J, Patra J, Zatonski W (2007). Comparing alcohol consumption in central and eastern Europe to other European countries. Alcohol Alcohol.

[CIT0042] Pridemore WA, Tomkins S, Eckhardt K, Kiryanov N, Saburova L (2010). A case-control analysis of socio-economic and marital status differentials in alcohol- and non-alcohol-related mortality among working-age Russian males. Eur J Public Health.

[CIT0043] Razvodovsky Y (2009). Alcohol poisoning and cardiovascular mortality in Russia 1956–2005. Alcoholism.

[CIT0044] Rehm J, Patra J, Baliunas D, Popova S, Roerecke M, Taylor B, Heggenhougen K, Quah S (2008). Alcohol, The burden of disease of.. International Encyclopedia of Public Health.

[CIT0045] Rehm J, Room R, Monteiro M, Gmel G, Graham K, Rehn N, Sempos CT, Frick U, Jernigan D, Ezzati M, Lopez AD, Rodgers A, Murray CJL (2004). Alcohol use. Comparative Quantification of Health Risks. Global and Regional Burden of Disease Attributable to Selected Major Risk Factors. Volume 1.

[CIT0046] Rehm J, Sulkowska U, Manczuk M, Boffetta P, Powles J, Popova S, Zatonski W (2007). Alcohol accounts for a high proportion of premature mortality in central and eastern Europe. Int J Epidemiol.

[CIT0047] Rehm J, Taylor B, Mohapatra S, Irving H, Baliunas D, Patra J, Roerecke M (2010). Alcohol as a risk factor for liver cirrhosis – a systematic review and meta-analysis. Drug Alcohol Rev.

[CIT0048] Rehm J, Mathers C, Popova S, Thavorncharoensap M, Teerawattananon Y, Patra J (2009). Global burden of disease and injury and economic cost attributable to alcohol use and alcohol-use disorders. Lancet.

[CIT0049] Savchuk SA, Kolesov GM, Nuzhnyi VP (2007). Chromatographic study of the chemical composition and potential toxicity of spirits and alcoholic beverages. J Anal Chem.

[CIT0050] Savchuk SA, Nuzhnyi VP, Kolesov GM (2006). Factors affecting the accuracy of the determination of diethyl phthalate in vodka, ethanol, and samples of illegal alcoholic products. J Anal Chem.

[CIT0051] Secretan B, Straif K, Baan R, Grosse Y, El Ghissassi F, Bouvard V, Benbrahim-Tallaa L, Guha N, Freeman C, Galichet L, Cogliano V (2009). A review of human carcinogens-Part E: tobacco, areca nut, alcohol, coal smoke, and salted fish. Lancet Oncol.

[CIT0052] Shield KD, Rehm M, Patra J, Sornpaisarn B, Rehm J (2011). Global and country specific adult per capita consumption of alcohol, 2008. Sucht.

[CIT0053] Solodun YuV, Klevno VA, Lelyukh TD, Maslauskaite LS, Yaverbaum AP, Ermolaeva NB, Zobnin YuV, Provado IP, Kuchina EV, Bogomolova IN (2008). Forensic-medical evaluation of toxic hepatitis associated with surrogate alcohol poisoning. Sud Med Ekspert.

[CIT0054] Stickley A, Razvodovsky Y, McKee M (2009). Alcohol mortality in Russia: A historical perspective. Public Health.

[CIT0055] Szücs S, Sárváry A, McKee M, Ádány R (2005). Could the high level of cirrhosis in central and eastern Europe be due partly to the quality of alcohol consumed? An exploratory investigation. Addiction.

[CIT0056] Tsisanova ES, Salomatin EM (2010). Forensic chemical investigation of alcohol-containing liquids doped with polyhexamethylene guanidine hydrochloride and diethylphthalate. Sud Med Ekspert.

[CIT0057] US EPA (2003). Diethyl phthalate (CASRN 84-66-2). Integrated Risk Information System. http://www.epa.gov/iris/subst/0226.htm.

[CIT0058] WHO (2003). Diethyl Phthalate. Concise International Chemical Assessment Document 52.

[CIT0059] WHO (2011a). Global Information System on Alcohol and Health (GISAH).

[CIT0060] WHO (2011b). Global status report on alcohol and health.

[CIT0061] Yushkov BG, Sencov VG, Bogdanov SI, Tyumenceva NV, Medvedeva SU, Danilova IG, Gafarova RK, Gette IF, Krohina NB, Zobnini UV, Zilberman FA (2011). Influence of polyhexamethyleneguanidine hydrochloride and CCl4 on structured-functional dates of the rat's liver in process of the shaping sharp hepatitis toxic genesis. Vest Ural Med Nauki.

[CIT0062] Zaigraev G, Haworth A, Simpson R (2004). The Russian model of noncommercial alcohol composition. Moonshine market: issues in unrecorded alcohol beverage production and consumption.

[CIT0063] Zaigraev G (2009). Alcoholism and drunkenness in Russia. Way out of the crisis situation. Sociol issled.

[CIT0064] Zaridze D, Brennan P, Boreham J, Boroda A, Karpov R, Lazarev A, Konobeevskaya I, Igitov V, Terechova T, Boffetta P, Peto R (2009). Alcohol and cause-specific mortality in Russia: A retrospective case-control study of 48 557 adult deaths. Lancet.

[CIT0065] Zatonski W, Manczuk M, Sulkowska U, HEM project team (2008). Closing the health gap in the European Union.

